# A critical appraisal evaluating the efficacy of extracorporeal shock wave therapy in coccydynia

**DOI:** 10.12968/ijap.2024.0066

**Published:** 2025-10-02

**Authors:** J Botton, C Hill, S Hill, S Jones, M Mccarney, M Goodall, JE Hill

**Affiliations:** Blackpool Teaching Hospitals NHS Foundation Trust; The University of Liverpool; University of Lancashire

## Abstract

Coccydynia is referred to as pain in the region of the coccygeal bone. It is usually managed conservatively with physiotherapy, education and medication. Symptoms can last a couple of weeks or up to five years. For severe cases, invasive interventions such as injections or surgery are currently the main options. The use of extracorporeal shock wave therapy (ESWT) is emerging as a successful, non-invasive treatment for a variety of musculoskeletal conditions. A systematic review by Nikouei et al., (2022) aimed to establish if ESWT was effective at alleviating pain for patients with coccydynia. This commentary provides a critical evaluation of the methods employed in this review and discusses the findings of the review in context to the four pillars of advanced practice: clinical practice, leadership and management, education, and research in physiotherapy.

## Introduction

Coccydynia has been referred to as pain that occurs in the region of the coccygeal bone or surrounding tissues (AntoniadisUlrich and Senyurt 2014). It represents less than 1% of non-traumatic back pain (NathanFisher and Roberts 2010) and has been documented to affect females more commonly (Foye 2017; Lirette et al. 2014). Symptoms can resolve within weeks with or without treatment, but some cases can become chronic (Lirette et al. 2014). Coccydynia can reduce the quality of life of patients who suffer with it (Foye 2017).

Coccydynia has been reported to be multifactorial; most patients report a previous history of trauma to the affected area, issues that have arisen following childbirth or biomechanical/ mobility issues of the coccyx itself (AntoniadisUlrich and Senyurt 2014; PatelAppannagari and Whang 2008). Treatment usually starts with conservative methods including the use of cushioning aids, physical therapy, medication and coccygeal manipulation (Lirette et al. 2014; PatelAppannagari and Whang 2008; SandrasegaramGupta and Baloch 2020). If such methods are not effective, more invasive treatment including corticosteroid injection and surgery may be considered (PatelAppannagari and Whang 2008), However, complications are associated with these invasive procedures (SandrasegaramGupta and Baloch 2020).

The use of extracorporeal shock wave therapy (ESWT) has been considered by some authors as a potentially effective and less invasive treatment to help reduce symptoms for those patients diagnosed with coccydynia (Lin et al. 2015; Marwan et al. 2017). Due to this growing body of evidence a systematic review and meta-analysis was undertaken by Nikouei et al., (2022) to assess the effectiveness of ESWT on coccydynia.

### Aim of commentary

This commentary aims to critically appraise and summarise the methods used within the systematic review by Nikouei et al., (2022) and discuss the findings of this review in context to the four pillars of advanced practice: clinical practice, leadership and management, education, and research in physiotherapy (Nikouei et al. 2022). Given their responsibilities in not only direct patient care but also in driving evidence-based research, educating others, and managing clinical resources, advanced practitioners must consider interventions like ESWT holistically, ensuring that implementation is considered with each of these domains.

### Critical appraisal and methods of Nikouei et al., (2022)

Utilizing the AMSTAR 2 critical appraisal tool for systematic reviews (Shea et al. 2017) only 9 out of 16 criteria were deemed satisfactory (see Table 1 critical appraisal and the methods used within the systematic review).

The primary areas of concern were centred around the data extraction process. The established gold standard for this procedure involves independent duplicate data extraction, as single data extraction has shown to introduce significant errors into review conclusions (Buscemi et al. 2006). The second area of concern was the absence of risk of bias (RoB) assessment of the included studies in the review (Viswanathan et al. 2018). Without this crucial information, it becomes challenging to gauge the confidence level in the estimate’s proximity to the true effect (Guyatt et al. 2011). Due to this lack of assessment of RoB the review also failed to analyse how the overall RoB may have affected the estimates presented. Similarly, there was also no assessment of the impact of heterogeneity on the review’s findings. Like RoB, this factor is essential for determining the certainty of an estimate (Guyatt et al. 2011). Furthermore, the review neglected to discuss or explore potential moderating factors which would be used in both identifying what factors are possibly important to optimise the effectiveness in the intervention and to explore issues of heterogeneity. Without these processes and statistical methods being undertaken, it is difficult to say what degree of certainty can be placed on the estimates presented within this review.

Concerning the search strategy, there was ambiguity regarding the use of the Consolidated Standards of Reporting Trials (CONSORT) statement, as it was suggested within the review that this guideline was employed to validate the search strategy. However, CONSORT is designed as a reporting standard for parallel-group randomized trials, leaving uncertainty about its specific application in this context. Similarly, the use of the Preferred Reporting Items for Systematic Reviews and Meta-Analyses (PRISMA) also lacked clarity in regard to how it was used. While PRISMA serves as a reporting standard (Page et al. 2021), its role as a tool to assess appropriateness of quality measurements of the meta-analysis is unclear. Another major concern was the decision to use only the before-and-after data from the randomized controlled trials (RCTs), rather than treating them as controlled trials with a comparator group for effect comparison. Ideally, the two RCTs should be meta-synthesized separately, with the before-and-after and retrospective studies used to verify the findings (Higgins et al. 2023). Instead, all four groups were combined as before-and-after studies, substantially reducing the certainty of the estimates presented. Moreover, there were concerns regarding the comprehensiveness of the search strategy, as there was no evidence of consultation with experts and no explanation provided for the exclusion of grey literature. However, this issue was considered of lesser concern given the specific context of the subject matter. Additionally, there was no indication that the funding sources of the studies included in the review were assessed. Transparency regarding funding is crucial, especially when the findings of a trial may have commercial implications. In summary, caution should be exercised when interpreting the findings of this systematic review, particularly concerning the comprehensiveness of the review methods and synthesis in addressing the research question of interest.

## Results of Nikouei et al., (2022)

The search strategy identified 2553 papers. After full screening, four studies were included (two RCTs and one before-and-after and one retrospective observational study). Two studies were from Iran and one each from Turkey and Taiwan. The meta-analysis examined the effect of ESWT on patients with coccydynia using a visual analogue scale (VAS) pain score (maximum pain score of 100). The main findings from the meta-analysis were;

- At 1 month after ESWT, the overall pooled mean VAS score decreased by 42.41 units (95% confidence interval [Cl]: −56.88 to −27.94, I^2^ = 86.96%)
- At 2 to 4 months, the overall pooled mean VAS score decreased by 41.01 units (95% CI: −46.98 to −35.04, I^2^ = 0%).
- At 6 to 12 months, the overall mean VAS score decreased by 50.13 units (95% CI of −67.33 to −32.94 I^2^ = 82.41%)

The meta-analysis revealed that ESWT had a significant effect on lessening pain in patients with coccydynia. The effect starting at the first month and increased during the 1-year follow-up, with the least pain occurring during the 6 to 12month period after using ESWT.

## Commentary

### Implications to practice

This review found a clinically important reduction in VAS score in people with coccydynia following ESWT. These effects were observable one month after treatment and appeared to improve during a one-year follow-up period. However, this meta-analysis had several limitations, including the low number of studies analysed, the lack of control groups used and no assessment of RoB of included studies. Furthermore, there was notable unexplained heterogeneity at one month and six to 12 months. These limitations diminish the reliability of the estimates presented in this review, impacting on the confidence with which these findings can be applied in advanced clinical practice.

Despite this reduced certainty in these estimates, ESWT is emerging as a safe and successful treatment option to improve patient pain and function for a range of musculoskeletal conditions including Achilles Tendinopathy (Feeney 2022), Greater Trochanteric Pain Syndrome (Harding et al. 2024; Heaver et al. 2021), Lateral Epicondylitis (Ibrahim et al. 2021; Yao et al. 2020), Pubis Osteitis (Schöberl et al. 2017) and Carpel Tunnel Syndrome (Li et al. 2020). For these pathologies within the studies, there was only minor side effects such as localised pain and swelling reported for the use of ESWT (Feeney 2022; Harding et al. 2024; Heaver et al. 2021; Ibrahim et al. 2021; Li et al. 2020; Schöberl et al. 2017). However, advanced practitioners should be mindful of contraindications, including anticoagulant disorders, acute infections, pregnancy, and direct application to growth plates, nerve tracts, or large vessels (De la Corte-Rodríguez et al. 2023). The National Institute for Health and Care Excellence (NICE) interventional guidelines endorse the use of ESWT for the treatment of Tennis Elbow, Plantar Fasciitis, Achilles Tendinopathy and Greater Trochanteric Pain Syndrome, however they recommend it be used with special arrangements for clinical governance, consent and audit or research due to inconsistent evidence for its efficacy (NICE 2009a; 2009b; 2011; 2016). Given this context, ESWT presents a viable non-invasive addition to conservative management approaches for coccydynia, offering advanced practitioners a broader spectrum of treatment options.

Regarding the specific application of ESWT the systematic review by Nikouei et al., (2022) recommend a dose of 2000 or 3000 impulses of shockwave with frequency of 5Hz and pressure of 2-4 bar, once weekly for 4 weeks to lesson pain in patients with coccydynia (Nikouei et al. 2022). This recommendation was based upon the parameters used in the included studies and provides a reference point for advanced practitioners when determining treatment protocols. Regarding the current NICE recommendations for the use of ESWT, the NICE interventional procedures guidance (NICE 2009a; 2009b; 2011; 2016) for Tennis Elbow, Plantar Fasciitis, Achilles Tendinopathy and Greater Trochanteric Pain Syndrome does not recommend specific treatment protocols for these conditions. They do advise that parameters can vary, this includes varying energy density or frequency of shockwaves (NICE 2009a; 2009b; 2011; 2016). Given that coccydynia is a disorder of the coccyx bone, it is important to mention that there is some evidence that higher energy ESWT provides more benefit in the treatment of disorders of bone (Tenforde et al. 2022), including avascular necrosis, non-union of fractures and stress injuries. It is proven to have anti-inflammatory, angiogenic, anti-oedema and trophic effects in the modification of cartilage and subchondral bone and bone remodelling (Al-Abbad et al. 2020; Tenforde et al. 2022).

### Management

When determining the stage at which ESWT should be introduced as an intervention in the management of coccydynia, advanced practitioners must evaluate evidence from a variety of sources. ESWT is mostly used in tendinopathy management, and therefore this is where most of the evidence exists. ESWT is offered when symptoms have not responded to conservative treatment such as physiotherapy, activity modification and pain relief (NICE 2016). Presumably this is because shockwave is more beneficial when the tendon is classed as degenerative, but also when conservative treatment has not been effective (van der Worp et al. 2013). However, tendinopathy loading programs can take up to 24 weeks to see significant improvements (Breda et al. 2021). A guideline for plantar heel pain (Morrissey et al. 2021) however recommended introducing ESWT approximately 4-6 weeks after usual care and education had been commenced. They did not recommend starting with ESWT as the evidence for stretching and education is far superior. The timelines reflect the time required for someone to respond to the core approach, but these can be modified based on the individual. However, there is not an abundance of evidence in patients with coccydynia as to whether conservative management is superior to ESWT. In the systematic review by Nikouei et al., (2022), results from an included RCT by Lin et al., (2015) indicated that both the group receiving ESWT and the group receiving usual care with electrotherapy experienced improvements in pain post-treatment (Lin et al. 2015). However, the ESWT group demonstrated more favourable improvements in disability scores at the eight-week mark.

Nikouei et al., (2022) specified participants with a minimum two-month history of coccydynia in their systematic review. Lin et al., (2015) rationalised that many cases of acute coccydynia will remit spontaneously in under two months and therefore will not require additional treatments. As we know symptoms of coccydynia can resolve in a couple weeks (Lirette et al. 2014), so there is an argument for starting treatment earlier than two months. Also, there is an argument for a combined approach of ESWT and conservative management. Burton (2022) suggested a combined approach is superior for the management of tendinopathy, therefore why not for coccydynia? This concept warrants further exploration and could serve as a valuable research direction to better inform advanced practitioners on optimal, individualized treatment strategies (Burton 2022).

### Education

Currently there are no internationally recognised pathway to become competent in applying ESWT for advanced practitioners (Tenforde et al. 2022). Typically, a train-the-trainer approach is used within practice. Advanced practitioners seeking to become proficient in ESWT may encounter barriers such as limited preceptor expertise, lack of available equipment, and insufficient literature outlining essential educational content (Bockbrader et al. 2019). Tenforde et al., (2022) described a core curriculum in ESWT application for clinicians who offer ESWT as a treatment modality, which includes six levels of competency with ‘key milestones’ to show competency (Tenforde et al. 2022). It has been suggested that clinicians should meet specific core competencies which include technical knowledge and procedural skills before completing specific treatment procedures on patients including ESWT (Tenforde et al. 2022), however specific clinical guidelines have not been established. It is recommended that a clinical framework for using ESWT should be established before using on patients to avoid complications or harm to patients (Bockbrader et al. 2019). These clinical guidelines should be taught face-to-face involving theory and practical-based elements including covering aspects of safety protocols and documentation of techniques and procedures. This should be followed up by supervised real-time clinical application with sufficient clinical feedback by clinical staff and patients (Bockbrader et al. 2019).

### Further research

Given the notable uncertainties surrounding the effectiveness estimates of ESWT for coccydynia, advanced practitioners should aim to conduct high-quality randomized controlled trials. Due to the substantial heterogeneity in the effects observed within the systematic review by Nikouei et al., (2022) it will be important for these controlled trials to assess possible important moderating factors such as frequency, pressure, duration and when the intervention is given within the care pathway for this condition. Furthermore, this review took a very narrow approach in regard to outcomes assessed, only looking at pain, and it is important that a more holistic outcome set be produced within future primary and secondary research. From a secondary research perspective, several key processes need improvement. These include double screening and data extraction processes, critical appraisal of the included studies, and preregistration of the protocol prior to starting the review. Additionally, further exploration of heterogeneity should be conducted when an adequate number of studies are identified, and the combination of RCTs should be prioritized to establish effect estimates, rather than relying solely on before-and-after data.

## Conclusions

The systematic review by Nikouei et al., (2022) aimed to assess the effectiveness of ESWT in alleviating pain for patients with coccydynia. The review found significant pain reduction with ESWT, which appears to increase over time. However, advanced practitioners should interpret these findings cautiously due to primary and secondary methodological issues within the limited evidence available. For other musculoskeletal conditions, ESWT has shown effectiveness with minimal adverse events reported, supporting its use as a secondary intervention following conservative treatments. Advanced practitioners may find it challenging to determine the optimal timing for ESWT in coccydynia, as current guidelines lack condition-specific recommendations. Although preliminary guidance exists for administering ESWT and identifying training needs, advanced practitioners are limited by the absence of detailed protocols specifically for coccydynia. Future research should focus on identifying key moderating factors, such as timing and dosage, and explore a wider range of clinically relevant outcomes to inform more nuanced, evidence-based recommendations tailored to advanced practice.

## Figures and Tables

**Table 1:** Critical appraisal of Nikouei et al., (2022) using the AMSTAR 2 critical appraisal tool

AMSTAR 2 items	Responses/Methods
Did the research questions and inclusion criteria for the review include the components of PICO?	**Yes** - the research questions and inclusion criteria for the review included the components of PICO (population, intervention, comparison, and outcome). The population was adults (>18 years old) with chronic coccydynia (>2 months history), the intervention was extracorporeal shock wave therapy (ESWT), the comparison was other treatments or no treatment, and the outcome was pain reduction measured by visual analogue scale (VAS) score for pain.
Did the report of the review contain an explicit statement that the review methods were established prior to the conduct of the review and did the report justify any significant deviations from the protocol?	**No** - the report of the review did not contain an explicit statement that the review methods were established prior to the conduct of the review and did not justify any significant deviations from the protocol.
Did the review authors explain their selection of the study designs for inclusion in the review?	**Yes** - the review authors explained their selection of the study designs for inclusion in the review. They included studies that had a reasonable study design to assess the effect of ESWT on coccydynia, such as randomized controlled trials (RCTs) or quasi-experimental studies.
Did the review authors use a comprehensive literature search strategy?	**Yes** - the review authors used a comprehensive literature search strategy. They searched electronic databases including Google Scholar, Scopus, ScienceDirect, ISI Web of Science, Embase, and PubMed, as well as some Iranian databases, using relevant keywords and synonyms. They also searched Current Contents and Cochrane Library for clinical trials registry and checked the references of review articles for additional studies.
Did the review authors perform the study selection in duplicate?	**Partial Yes** - it is indicated that all steps of the search strategy were undertaken by two reviewers, but it is unclear exactly what this means and if this was carried out independently.
Did the review authors perform data extraction in duplicate?	**No** - it is not clear as to the exact number of reviewers who carried out data extraction.
Did the review authors provide a list of excluded studies and justify the exclusions?	**No** - the review authors did not provide a list of excluded studies or justify the exclusions. They only reported the number of studies that did not meet the inclusion criteria at each stage of the screening process but did not name or describe them.
Did the review authors describe the included studies in adequate details?	**Partial Yes** – the review authors described the included studies in adequate details. They provided information on the first author’s name, publication year, country, study design, sample size, participants’ characteristics, ESWT parameters, and mean VAS score before-and-after ESWT. However further information could have been provided regarding the control group.
Did the review authors use a satisfactory technique for assessing the risk of bias (RoB) in the individual studies that were included in the review?	**No** – the review authors did not use a satisfactory technique for assessing the RoB in the individual studies that were included in the review. They did not report any formal quality assessment tool or criteria to evaluate the methodological quality of the studies, such as randomization, allocation concealment, blinding, attrition, or reporting bias.
Did the review authors report on the sources of funding for the studies included in the review?	**No** - the review authors did not report on the sources of funding for the studies included in the review. They did not mention whether the studies received any financial support or sponsorship from any organization or institution.
If meta-analysis was performed did the review authors use appropriate methods for statistical combination of results?	**No** - they calculated the mean changes of VAS score and its 95% confidence intervals for each study and pooled them using random or fixed effects models depending on the heterogeneity test. They also performed subgroup analysis based on the follow-up duration of the studies. The unusual decision was made to only compare before-and-after data of the intervention group rather than assessing the effect compared to the control.
If meta-analysis was performed did the review authors assess the potential impact of RoB in individual studies on the results of the meta-analysis or other evidence synthesis?	**No** - the review authors did not assess the potential impact of RoB in individual studies on the results of the meta-analysis. They did not perform any sensitivity analysis or meta-regression to explore the effect of study quality or other covariates on the pooled estimate.
Did the review authors account for RoB in individual studies when interpreting/discussing the results of the review?	**No** - because they did not carry out a RoB assessment, they did not discuss the findings in context to this.
Did the review authors provide a satisfactory explanation for and discussion of, any heterogeneity observed in the results of the review?	**Yes** - the review authors provided a satisfactory explanation for and discussion of, any heterogeneity observed in the results of the review. They reported the I-squared statistic.
If they performed quantitative synthesis did the review authors carry out an adequate investigation of publication bias (small study bias) and discuss its likely impact on the results of the review?	**Yes partial** - they only performed the Egger’s test to detect publication bias, but did not provide any graphical representation, such as a funnel plot or a contour-enhanced funnel plot, to visualize the asymmetry of the studies.
Did the review authors report any potential sources of conflict of interest, including any funding they received for conducting the review?	**Yes** - the review authors reported any potential sources of conflict of interest, including any funding they received for conducting the review. They stated that they had no conflicts of interest and that the review was supported by the Iran University of Medical Sciences.
